# Clinical outcomes, MRI evaluation and predictive factors of indirect decompression with lateral transpsoas approach for lumbar interbody fusion: a multicenter experience

**DOI:** 10.3389/fsurg.2023.1158836

**Published:** 2023-04-03

**Authors:** Salvatore Petrone, Marco Ajello, Nicola Marengo, Marco Bozzaro, Alessandro Pesaresi, Mario Allevi, Alessandro Fiumefreddo, Federica Denegri, Maurizio Cogoni, Andrea Garnero, Fulvio Tartara, Giuseppe Di Perna, Daniele Armocida, Alessandro Pesce, Alessandro Frati, Francesco Zenga, Diego Garbossa, Fabio Cofano

**Affiliations:** ^1^ Spine Surgery Unit, Humanitas Gradenigo Turin, Turin, Italy; ^2^ Neurosurgery Unit, University of Turin Department of Neurosciences Rita Levi Montalcini, Turin, Italy; ^3^ Neurosurgery Unit, AOU Città della Salute e della Scienza, Turin, Italy; ^4^ Neuroradiology Unit, AOU Città della Salute e della Scienza, Turin, Italy; ^5^ Spine Surgery Unit, Casa di Cura Città di Bra, Bra, Italy; ^6^ Neurosurgery Unit, Department of Human Neuroscience, University Sapienza of Rome, Rome, Italy; ^7^ Neurosurgery Unit, Ospedale Santa Maria Goretti, Latina, Italy; ^8^ Skull Base and Pituitary Surgery Unit, AOU Città Della Salute e Della Scienza, Turin, Italy

**Keywords:** indirect decompression, LLIF, XLIF, spondylolisthesis, predictive factors

## Abstract

**Introduction:**

Evaluating the effects of indirect decompression obtained through lateral lumbar interbody fusion (LLIF) by clinical improvements and radiological parameters on MRI scans. Identifying predictors of better decompression and clinical outcome.

**Materials and methods:**

From 2016 to 2019, patients who underwent single- or double-level indirect decompression LLIF were consecutively reviewed. Radiological signs of indirect decompression were evaluated in preoperative and follow-up MRI studies and were subsequently correlated to clinical data, expressed as axial/radicular pain (VAS back/leg), index of disability (Oswestry Disability Index) and clinical severity of lumbar stenosis (Swiss Spinal Stenosis Questionnaire).

**Results:**

72 patients were enrolled. The mean follow-up was 24 months. Differences in vertebral canal area (*p* < 0.001), height of the foramina (*p* < 0.001), thickness of the yellow ligament (*p* = 0.001) and anterior height of the interbody space (*p* = 0.02) were observed. Older age (*p* = 0.042), presence of spondylolisthesis (*p* = 0.042), presence of intra-articular facet effusion (*p* = 0.003) and posterior height of the implanted cage (*p* = 0.020) positively affected the increase of the canal area. Change in root canal area (*p* < 0.001), height of the implanted cage (*p* = 0.020) and younger age (*p* = 0.035) were predictive factors of root pain relief, while increased vertebral canal area (*p* = 0.020) and height of the interbody fusion cage (*p* = 0.023) positively affected the severity of clinical stenosis.

**Conclusions:**

LLIF indirect decompression showed both clinical and radiological improvements. Presence and degree of spondylolisthesis, presence of intra-articular facet effusion, age of the patient and height of the cage were predictive factors of major clinical improvements.

## 1. Introduction

The lateral lumbar interbody fusion (LLIF) technique represents a minimally invasive alternative for the treatment of degenerative lumbar disease, reducing potential vascular, visceral, and sympathetic chain complications of pure anterior approaches while avoiding the morbidity of the traditional posterior approach (1).

Even if many types of new minimally invasive approaches in spine surgery showed to be safe and valuable options in spine surgery (2–6), the lateral approach to the spine retains the advantage of wide access to the intervertebral disc and the possibility of placing larger cages to maximize contact with cortical bone, preserving both the anterior and the posterior longitudinal ligaments. Furthermore, the high and large cage also allows indirect neural decompression of the spinal canal and foramen by restoring the height of the disc and stretching the ligaments (7). Moreover, it is a very flexible technique that can be used in a large variety of patients (e.g., obese) without a greater risk of complications (8).

Several studies demonstrated the effect of indirect LLIF decompression through the improvements of radiographic parameters such as the dura mater cross-sectional area or foraminal area; however, in literature, some cases which required second surgery due to insufficient indirect decompression leading to central residual canal stenosis or lateral bone recess stenosis are reported (9). So, up to now, the efficacy of indirect decompression in LLIF is still controversial.

The first goal of this study was to evaluate the effect of LLIF indirect decompression by pre- and postoperative clinical variations. The second goal was to compare radiological parameters on pre- and post-surgical MRI scans. The third objective was to find a correlation between indirect decompression and mid-term clinical improvements, identifying predictors of better decompression.

## 2. Materials and methods

In this multicenter study, patients treated with LLIF from 1 July 2016 to 1 November 2019 were retrospectively identified and then enrolled at authors' institutions for radiological and clinical investigation. Informed consent for the collection of data and an MRI scan for scientific purposes was collected. Given the retrospective nature of the study, no institutional approval was required.

Only transpsoas interbody fusions with indirect decompression technique on single or double level for central stenosis caused by degenerative disc disease, spondylolisthesis, degenerative scoliosis and adjacent segment disease were included for this study. All recruited patients complained of low back pain and bilateral claudication with or without specific nerve root pain, unresponsive to conservative medical therapies for more than 8 weeks. Besides, patients who received a direct decompression procedure on the vertebral canal were excluded, as well as patients with unilateral nerve root pain without claudication or history of infections, tumors or trauma.

All clinical and radiological evaluations were performed retrospectively on the final sample.

### 2.1. Surgical technique

The patient is sedated and intubated from the supine position, and then placed in lateral decubitus according to the preoperative planning of the side to be exposed after electrodes application for neuromonitoring. Afterward, lateral lumbar bending is achieved through surgical table flexion and the access point is verified based on lateral x-ray images.

The detachment of subcutaneous and muscular tissues (external oblique, internal and transverse muscle) is performed with the fingers or with dissecting scissors until the retroperitoneal fat is reached.

The dilator and the stimulation probe are guided toward the surface of the psoas muscle, perpendicular to the disc and gently inserted to dilate the fibers of the psoas muscle. Progressively larger dilators are inserted, associated with neurostimulation to avoid unsafe entry zones. Then, the retractor is positioned with the knobs facing rearwards and fixed to an arm previously mounted on the table. The light sources are hooked onto the blades.

Annulotomy is performed by making two longitudinal and two transverse incisions to form a rectangle with a scalpel; the nucleus pulposus and the cartilage of the vertebral plate are removed, and the annulus of the contralateral side is opened to allow parallel distraction of the disc space and avoid deformities.

Progressive sized trial cages are inserted, and the length of the trial cage is compared with that of the vertebral plate and the height of the implant using fluoroscopic guidance. Once the definitive cage has been chosen, the fenestrated part is filled with autologous or synthetic bone and then implanted with gentle thrusts into the space prepared. Then, posterior instrumentation with open or percutaneous transpedicular screwing is performed.

### 2.2. Clinical evaluation

Patient electronic medical records were reviewed for images, clinical notes, and clinic evaluations. Enrollment of patients included the following data: sex, age, height, Body Mass Index (BMI), indication for surgery, any assumption of antiplatelet agents, anticoagulants or coagulation pathologies and smoking habit. The variables regarding the surgical procedure were the number of levels treated, dimensions and lordosis of the interbody fusion cage, dimensions of the pedicle screws and type of posterior approach.

Preoperative data regarding radicular pain (VAS leg), axial pain (VAS back), the index of disability (Oswestry Disability Index—ODI) and the index of clinical severity of lumbar stenosis (Swiss Spinal Stenosis Questionnaire) were collected. Duration of the surgical procedure, intra- and peri-operative complications, verticalization day, days of hospitalization, analgesic drugs taken at discharge were collected as well as VAS leg/back, ODI, Swiss Spinal Stenosis Questionnaire data at discharge, at 1 month after surgery and at least 6 months after the surgical procedure.

### 2.3. Radiological evaluation

All preoperative lumbosacral MRI studies of patients undergoing LLIF surgery with indirect decompression were collected. All retrospectively recruited patients underwent lumbosacral MRI post-postoperatively, performing 3D volumetric T1-weighted and T2-weighted sequences using a 1.5 T scanner. In addition, we performed a sagittal 2D STIR TSE sequence of the lumbar spine in order to assess bone marrow edema. Sequence parameters are reassumed in Table 1.

**Table 1:** MRI sequences parameters.

MRI sequences	TR (ms)	TE (ms)	Slice thickness (mm)	FOV (mm)	Bandwidth (Hz)	Dimension
T1W Space	500	17	1.50	256	610	3D
T2W Space	1500	125	1.50	256	558	3D
STIR TSE	4650	73	4.00	320	169	2D

On preoperative and postoperative MRI were measured: (1) maximum axial vertebral canal area at the index level; (2) left and right foramen area; (3) left and right foramen height; (4) left and right yellow ligament thickness; (5) anterior and posterior disc height; (6) degree of spondylolisthesis; (7) presence of facet joint effusion. On postoperative MRI we measured the distance between the interbody cage and the posterior somatic wall of the inferior vertebral body.

All measurements were performed by two independent neuroradiologists, using the digital visualization system Synapse 3D. All measurements were obtained from the averages of the measurements of the two individual observers.

### 2.4. Statistical analysis

The distribution of continuous variables was preliminarily evaluated by the Saphiro-Wilk test. Preoperative and postoperative variables were compared by paired Student's T test or Wilcoxon test as appropriate based on the distribution of variables. Linear regression models were used to identify predictive variables of radiological (percentage increase in the spinal canal area) and clinical outcome (VAS leg and Swiss Scale). Based on the univariate analyzes and the data present in the literature, the variables to be included in the multivariate model were selected. The confidence interval used was 95%. Inter-rater agreement was evaluated by kappa statistics. The statistical analysis was carried out using the “IBM SPSS Statistics 27” and “GraphPad Prism version 8.0.0 for Windows, GraphPad Software, San Diego, California USA, www.graphpad.com” software.

## 3. Results

After review of medical records, 171 patients undergoing LLIF procedures were initially identified. 77 patients undergoing lateral fusion with direct decompression were excluded. 6 patients who underwent a surgical procedure for lumbar spine fracture and 4 patients for metastatic lesions were excluded. The remaining sample was contacted by telephone to verify availability to undergo a medical examination and postoperative MRI study. 12 patients were untraceable or unwilling to participate in the study.

72 consecutive patients treated with LLIF technique and indirect decompression were considered. 41.7% of them were male. The mean age was 61.2 years (range 30.3–82.0) and the mean BMI was 24.4 (range 18.8–33.3). Smokers were 28.6%. About half of the patients were treated for degenerative spondylolisthesis, 40.5% for stenosis caused by wide protrusion disc and 9.5% for *de novo* scoliosis. A total of 86 treated spine levels were considered. All the data are summarized in Table 2.

**Table 2:** Population characteristics.

Characteristics	Value
**Gender**	
Male	41.7% (30)
Female	58.3% (42)
**Age (years)**	
Mean (range)	61.2 (30–82)
**BMI**	
Mean (range)	24.4 (18.8–33.3)
**Surgical Indication (%)**	
Stenosis	40.5%
Spondylolisthesis	50%
Degenerative scoliosis	9.5%
**Symptoms**	
Low back pain	100%
Neurogenic claudication	100%
Additional root pain	87.5%
**Treated levels**	
Total	86
L1-L2	5.8%
L2-L3	9.4%
L3-L4	26.7%
L4-L5	58.1%
**Posterior instrumentation technique**	
Open	41 (57%)
Percutaneous	31 (43%)
Follow-up (months)	
Mean (range)	24 (7.2–56.4)

The most treated level was L4-L5 (58.1%), followed by L3-L4 (26.7%). The material of the interbody fusion cage was Titanium in 57% and PEEK (Polyetheretherketone) in the remaining 43% of cases. In about half of the cases, the interbody fusion cages had a height of 12 mm (55.8%), while in 37.2% of 10 mm. The lordosis of the cage was equal to 10° in 55.8%, 8° in 32.6% and parallel (0°) in the remaining 11.6%. The placement of pedicle screws was performed with the percutaneous technique in 61.9% of cases, while in the other cases with the open technique according to surgeons' preferences (Table 2). Even in the open procedures, no direct decompression of the vertebral canal was performed.

The mean follow-up period was 24 months (range 7.2–56.4).

### 3.1. Clinical outcomes

**Figure 1:**
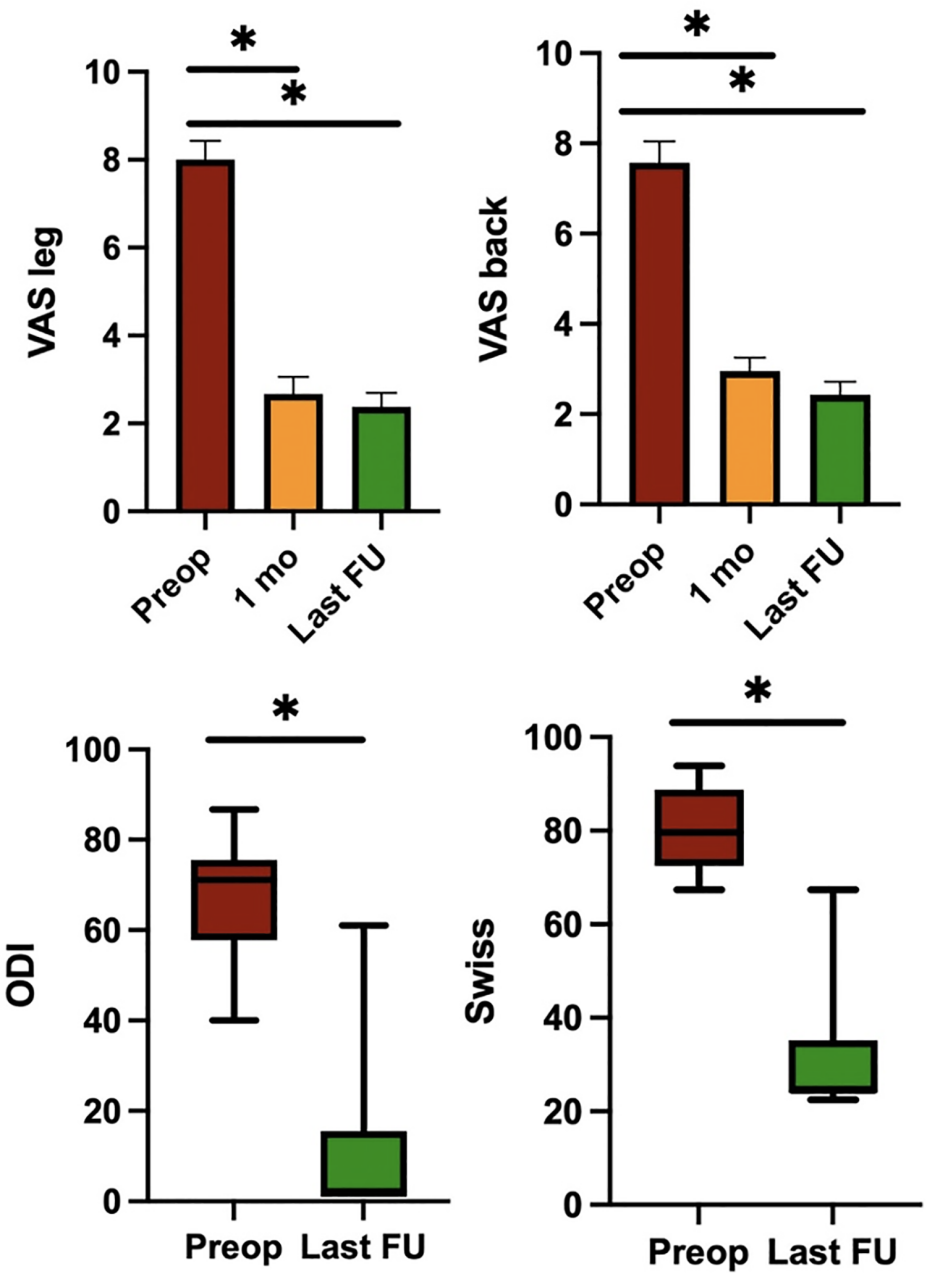
Pre- and post-operative clinical outcomes. The mean values of VAS leg, VAS back, ODI and Swiss Spinal Stenosis Questionnaire. Note: *P < 0.05; VAS, Visual Analog Scale; ODI, Oswestry Disability Index.

Preoperative mean values of the VAS back and VAS leg were 7.43 and 8.00 respectively, while VAS back and VAS leg at the time of discharge were 4.43 and 3.81. One month after the surgical procedure and at the last follow-up, VAS back and VAS leg were 2.95 and 2.67, and 1.95 and 1.85 respectively. All clinical variations of axial and radicular pain at the different times of data collection were statistically significantly different (*p* < 0.001). The degree of disability expressed by the Oswestry Disability Index showed a significant reduction from 66.7% preoperatively to 10.9% at the last follow (*p* < 0.001, SD 15.8). The improvement in the disability index was 55.8% in two years. The clinical severity of lumbar stenosis expressed by the Swiss Spinal Stenosis Score was 80.7% in the preoperative period, while it was 32.0% at the last follow-up (*p* < 0.001, SD 12.7). Clinical outcomes are graphically reported in Figure 1.

Postoperative complications recorded were iliopsoas weakness in 15.3% of cases (all temporary deficits); two cases of hypoparesthesia along the anterior part of the thigh (one of which was permanent). A single intraprocedural complication occurred in our series: rupture of an interbody PEEK fusion cage during its placement. No re-operations were performed due to insufficient decompression.

### 3.2. Radiological outcomes

**Figure 2:**
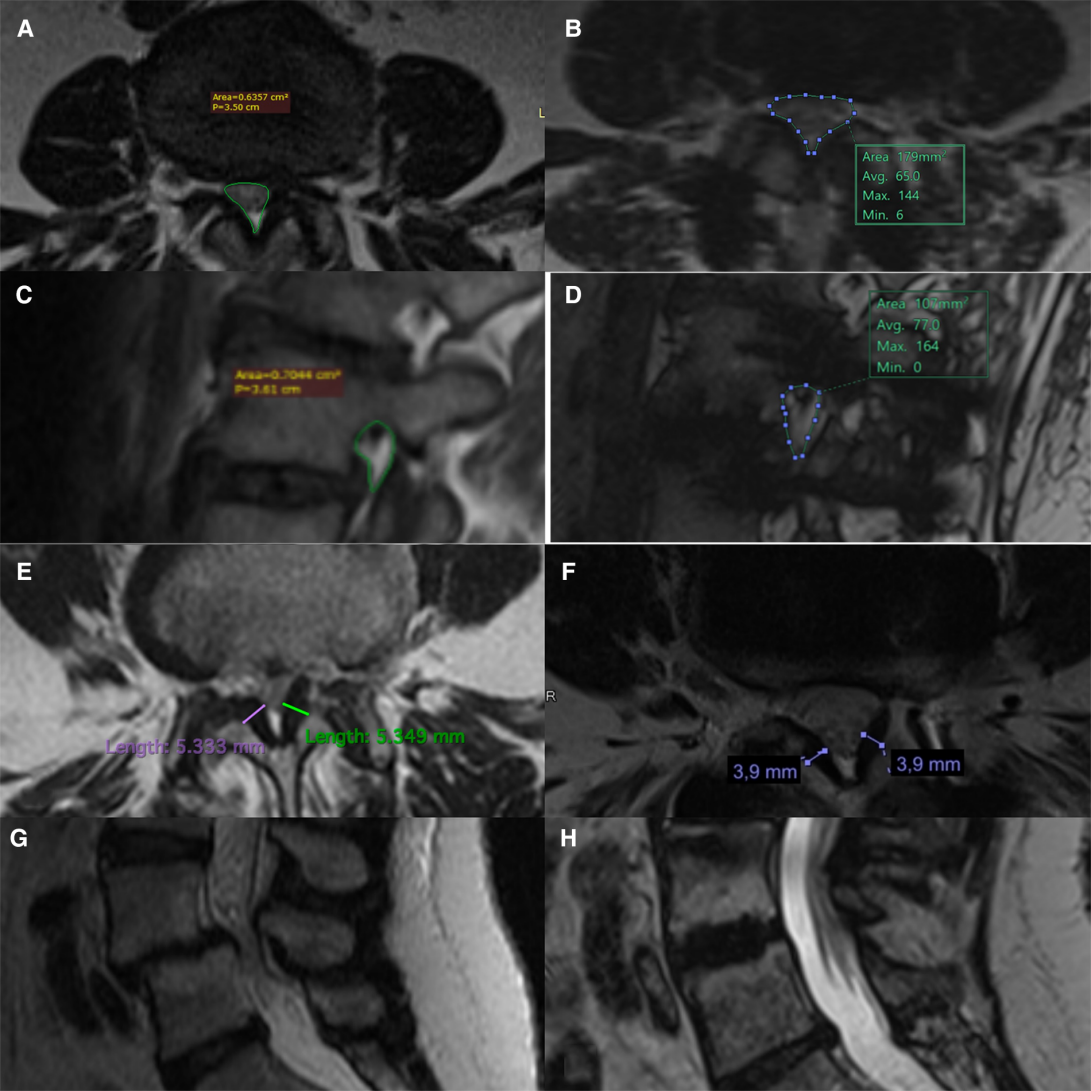
Pre- and post-operative comparison on MRI scans. Measurement of pre and post-op central canal area (**A,B**), foramen area (**C,D**), thickness of the yellow ligament (**E,F**). Example of reduction of degenerative spondylolisthesis through indirect LLIF decompression technique (**G,H**).

Considering preoperative and postoperative measurements on T2-weighted MRI, the difference in vertebral canal area was 70.8 mm² ± 28.7 with an increase of 68% (137.5 mm² ± 62.6 vs. 204.8 mm² ± 65.0, *p* < 0.001). The difference in area of the right foramen was 41.9 mm² ± 27.9 with an increase of 68% (77.3 mm² ± 26.2 vs. 116.5.7 mm² ± 30.7, *p* < 0.001), while that of the left foramen 42.0 mm² ± 27.3 with an increase of 60% (81.7 mm² ± 24.6 vs. 121.2 mm² ± 27.3, *p* < 0.001). The difference in height of the right foramen was 4.0 mm ± 3.6 with an increase of 32% (13.7 mm ± 3.2 vs. 17.7 mm ± 15.1, *p* < 0.001), while that of the left foramen was equal to 3.5 mm ± 3.0 with an increase of 29% (14.4 mm ± 14.5 vs. 17.7 ± 15.1, *p* < 0.001). The difference of thickness of the yellow ligament was 0.9 mm ± 0.9 with a reduction of 18% (4.3 mm ± 0.9 vs. 3.4 mm ± 0.9, *p* = 0.001). The change in the anterior height of the interbody space was 1.5 mm with an increase of 20% resulting statistically significant (7.9 mm ± 2.6 vs. 9.4 ± 2.9, *p* = 0.02), while the posterior one showed a difference of 0.4 mm with an increase of 10% (5.4 mm ± 6.1 vs. 5.8 mm ± 6.1, *p* > 0.05) (Figure 2). Inter-rater reliability between the two neuroradiologists was very good (k = 0.817).

### 3.3. Radiological and clinical predictors

On multivariate analysis, older age (*p* = 0.042), the presence of spondylolisthesis (*p* = 0.042), the presence of intra-articular facet effusion (*p* = 0.003) and the posterior height of the interbody fusion cage (*p* = 0.020) resulted to be the variables that positively affect the increase of the canal area (Table 3).

**Table 3:** Multivariate analysis of variables affecting the increase of the canal area between pre- and post-operative measurements.

Variables	B	St. Error	Beta	t	*p*-value	95% CI for Exp(B)	
						Lower	Upper
Age	1.406	0.664	0.386	2.117	**0**.**042**	0.051	2.762
BMI	1.456	2.259	0.098	0.645	0.524	−3.151	6.064
Facet effusion	62.124	19.547	0.603	3.178	**0** **.** **003**	22.257	101.991
Spondylolisthesis	29.450	13.890	0.339	2.120	**0** **.** **042**	1.121	57.779
Anterior height of the interbody space	−2.509	4.844	−0.143	−0.518	0.608	−12.388	7.370
Posterior height of the interbody space	9.843	5.650	0.472	1.742	0.091	−1.681	21.367
Cage to posterior body wall distance	3.583	3.171	0.312	1.130	0.267	−2.884	10.049
Height of the cage	−15.621	6.391	−0.419	−2.444	**0** **.** **020**	−28.656	−2.586

Regarding the clinical outcome in terms of radicular pain variation expressed as the difference between the preoperative and last postoperative VAS leg, our analysis showed that the change in canal area (*p* < 0.001), the height of the implanted interbody cage (*p* = 0.020) and younger age (*p* = 0.035) were strongly correlated with the improvement of root pain on multivariate analysis (Table 4).

**Table 4:** Multivariate analysis of variables correlated with the improvement of root pain (VAS leg).

Variables	B	St. Error	Beta	t	*p*-value	95% CI for Exp(B)	
						Lower	Upper
Age	−0.078	0.035	−0.372	−2.196	0.035	−0.150	−0.006
BMI	0.006	0.120	0.007	0.049	0.961	−0.237	0.249
Facet effusion	−0.894	1.073	−0.151	−0.833	0.411	−3.076	1.289
Spondylolisthesis	0.380	0.810	0.065	0.469	0.642	−1.269	2.028
Height of the cage	1.108	0.301	0.518	3.685	**0**.**001**	0.496	1.720
Increase in vertebral canal area (%)	0.036	0.009	0.626	4.036	**0**.**000**	0.018	0.054

Finally, the improvement of vertebral canal area (*p* = 0.020) and the height of the interbody fusion cage (*p* = 0.023) resulted to be able to significant modify the clinical severity of lumbar stenosis, expressed as a percentage difference of the Swiss Spinal Stenosis Questionnaire, on multivariate analysis (Table 5).

**Table 5:** Multivariate analysis of variables improving the clinical severity of lumbar stenosis (Swiss spinal stenosis questionnaire).

Parameters	B	St. Error	Beta	t	*p*-value	95% CI for Exp(B)	
						Lower	Upper
Age	−0.59	0.178	−0.608	−0.335	0.740	−0.421	0.302
BMI	0.029	0.600	0.008	0.049	0.961	−1.191	1.249
Spondylolisthesis	1.438	4.064	0.059	0.354	0.726	−6.831	9.706
Height of the cage	3.600	1.508	0.401	2.387	**0**.**023**	0.531	6.669
Facet effusion	−1869	5.382	−0.075	−0.347	0.731	−12.818	9.080
Increase in vertebral canal area (%)	0.109	0.045	0.454	2.445	**0**.**020**	0.018	0.200

## 4. Discussion

LLIF has certainly extended its indications over the past two decades, effectively changing the limits of spinal surgery. The main surgical indication is certainly concerning degenerative pathology of the lumbar spine, being LLIF a minimally invasive decompression/fusion tool for numerous conditions such as lumbar stenosis, spondylolisthesis, scoliosis, bulging disc in patients with a very wide and variable age range. Given this, many papers have been published dealing also with the use of LLIF for surgical revision procedures of previous stabilizations and for the treatment of adjacent disc disease (10), for the surgical treatment of thoracolumbar fracture (11, 12), spondylodiscitis (13, 14), and, more recently, for the surgical treatment of neoplastic lesions of the dorsal and lumbar spine (15, 16). Also, intraoperative neurophysiological monitoring helps to drastically reduce the risks of neurological complications related to the surgical procedure (17).

Our results showed that there is a progressive improvement of axial pain (VAS back) and radicular pain (VAS leg), as well as ODI scores and clinical severity of lumbar stenosis and that these differences persist years after the surgical procedure. Clinical benefits of indirect decompression on axial pain components and foraminal radicular pain have been widely consolidated in the literature, while the results on the pain component attributable to central stenosis of the vertebral canal are still poor in strong literary evidence. In this study, VAS leg and Swiss Spinal Stenosis scores obtained seem to confirm the benefits of indirect decompression of the central canal with the ligamentotaxis technique (18).

Regarding radiological outcomes, the use of a postoperative MRI study with volumetric acquisitions has allowed the best definition of the one-dimensional components and areas of interest (19, 20). The foraminal areas and the anterior height of the index intervertebral space differed significantly after surgery: the increase in foraminal heights is around 30%, that of the foraminal areas around 60% and that of the anterior intervertebral space is around 20%. These data are in agreement with other studies (21–24).

The increase in the posterior height of the disc space was quantified at about 10% but did not reach a statistical significance although satisfactory outcomes. This could be explained by many factors. One should consider the hypothesis that after two years fusion processes limited MRI analysis of posterior disc height because of the formation of bone bridges and artifacts. Furthermore, subclinical subsidence phenomena could have occurred, limiting the mean of the variation. Subsidence represents one of the main risks of clinical failure after indirect decompression, burdened by lower rate of fusion, lower maintenance of indirect decompression and higher rate of re/operation (25). It is therefore possible that over time a subgroup of the sample lost a part of the distraction of the vertebral plates.

The mean value of the increasing of the area of the central canal was approximately 70 mm² (+ 68%) and this difference was statistically significant (*p* < 0.001). The increase of about 30% in the canal area is confirmed in the literature in several studies (7, 11, 18, 20, 26–30). Similarly, the differences in terms of millimeters of the thickness of the yellow ligament were statistically significant between pre- and post-operative (*p* = 0.001): from a radiological point of view these data are of fundamental importance in determining the effectiveness of indirect decompression and tension on ligament structures (ligamentotaxis). The significant reduction in the thickness of the yellow ligament is reported in relatively few studies (31) and represents the most direct measurement of the ligamentotaxis principle.

The second part of the study aimed to identify any predictive factors of clinical and radiological outcomes.

Regarding radiological outcomes, the predictive factors for the increase of the area of the vertebral canal were older age, the presence of spondylolisthesis, the presence of intra-articular effusion and the height of the interbody fusion cage. Degenerative spondylolisthesis represents one of the main indications for LLIF with an indirect technique because the restoration of the correct alignment of the posterior wall allows for the largest increases in canal area. Moreover, increasing age was associated with major variations in canal area: this could be explained considering that the progress of vertebral degeneration in the preoperative areas is significantly lower in younger patients. The presence of facet joint effusion would seem to be correlated with a better radiological outcome as it would represent a sign of mechanical instability both on the antero-posterior and rotational plan (32). The size of the interbody fusion cage was found to be inversely related to the percentage increase of the central canal area. This could be justified as intervertebral discs with more advanced degeneration states (e.g., Pfirrmann IV) allow the greatest variations of the radiological parameters even with smaller cages compared to discs with moderate/mild degeneration.

From the analysis, the variables of the percentage change in the spinal canal area and the height of the implanted cage emerged as predictors of the radicular pain in lower limbs, expressed as VAS leg. Also, younger age was correlated with a better clinical outcome in terms of VAS leg. This is attributable to a lower capacity for recovery and regeneration of the peripheral nerve tissues and the overlap of different pain generators affecting the lower limbs in elderly patients (e.g., coxarthrosis, gonarthrosis).

Finally, the percentage change in the canal area and the height of the interbody fusion cage were also predictive variables of improvement of lumbar stenosis, evaluated as the percentage change in the Swiss Spinal Stenosis Questionnaire.

No patients required a second surgery for direct decompression during the two years of follow-up, therefore it was hard to investigate negative factors affecting the clinical results. In the literature, some conditions limiting clinical outcomes are reported. According to the study by Wang et al. the degenerative element of bony lateral recess stenosis represents a real limitation of the indirect decompression technique obtained with LLIF (7).

This study therefore presents possible predictive tools for both radiological and clinical outcomes. The results are largely in agreement with other studies in the literature (30). In the study by Walker et al. 73 patients were examined for the identification of predictive factors of radiological outcome. The variables most able to influence the variation of canal area were the lower BMI, the presence of spondylolisthesis and the lower posterior height of the intervertebral space. The clinical follow-up was 1 year while the post-operative evaluation with lumbosacral spine MRI was performed on the 1st or 2nd postoperative day (33). In the present study, the latest clinical and radiological evaluations correspond to a mean follow-up of 2 years, with a minimum follow-up of 7 months. This data makes it possible to attribute greater weight to the predictive factors identified which may also have a value in the medium term.

## 5. Limitations

This study has several limitations, being its retrospective nature the most important. However, the goal of the study was not to obtain an algorithm for the use of indirect decompression with LLIF surgery according to patients' characteristics. This paper aimed to highlight a strong association between LLIF indirect decompression and clinical/radiological outcomes, in order to underline preoperative features predicting better results. The sample size is another limitation, but the number of involved patients allowed a proper statistical evaluation.

The follow-up after the first six months, consisting of clinical evaluation and MRI study, was variable over time and therefore not homogeneous for all the patients.

## 6. Conclusions

The indirect decompression obtained by the lateral transpsoas approach is a valid tool for the surgical treatment of many degenerative conditions of the lumbar spine. Clinical and radiological improvements were confirmed in the medium-term follow-up being effective not only on foraminal but also for central stenosis symptoms. The presence and degree of spondylolisthesis, the presence of intra-articular facet effusion, the age of the patient and the height of the interbody fusion cage were predictive factors of major clinical improvements. The significant reduction in the thickness of the yellow ligament represents the direct measurement of the ligamentotaxis technique. Further studies are needed for the large-scale validation of the positive and negative predictors of indirect decompression LLIF with a particular interest in the possible negative predictive significance of recessual bone stenosis.

## Data Availability

The raw data supporting the conclusions of this article will be made available by the authors, without undue reservation.
